# A chloroquine-induced macrophage-preconditioning strategy for improved nanodelivery

**DOI:** 10.1038/s41598-017-14221-2

**Published:** 2017-10-23

**Authors:** Joy Wolfram, Sara Nizzero, Haoran Liu, Feng Li, Guodong Zhang, Zheng Li, Haifa Shen, Elvin Blanco, Mauro Ferrari

**Affiliations:** 1 Department of Nanomedicine, Houston Methodist Research Institute, Houston, TX 77030 USA; 2 Department of Transplantation, Mayo Clinic, Jacksonville, FL 32224 USA; 3 Applied Physics Graduate Program, Rice University, Houston, TX 77005 USA; 4 Department of Cell and Developmental Biology, Weill Cornell Medicine, New York, NY 10065 USA; 5 Department of Medicine, Weill Cornell Medicine, Weill Cornell Medicine, New York, NY 10065 USA

## Abstract

Site-specific localization is critical for improving the therapeutic efficacy and safety of drugs. Nanoparticles have emerged as promising tools for localized drug delivery. However, over 90% of systemically injected nanocarriers typically accumulate in the liver and spleen due to resident macrophages that form the mononuclear phagocyte system. In this study, the clinically approved antimalarial agent chloroquine was shown to reduce nanoparticle uptake in macrophages by suppressing endocytosis. Pretreatment of mice with a clinically relevant dose of chloroquine substantially decreased the accumulation of liposomes and silicon particles in the mononuclear phagocyte system and improved tumoritropic and organotropic delivery. The novel use of chloroquine as a macrophage-preconditioning agent presents a straightforward approach for addressing a major barrier in nanomedicine. Moreover, this priming strategy has broad applicability for improving the biodistribution and performance of particulate delivery systems. Ultimately, this study defines a paradigm for the combined use of macrophage-modulating agents with nanotherapeutics for improved site-specific delivery.

## Introduction

The circulatory system is frequently exploited for drug delivery purposes, as it is often challenging to reach diseased cells through local interventions. The major drawback of systemic drug administration is the development of side effects due to an inability to achieve site-specific delivery. The occurrence of side effects necessitates adherence to a specific drug concentration range, which is usually insufficient to obtain curative therapeutic efficacy in aggressive and drug-resistant diseases. Therefore, treatment of disease can fundamentally be viewed as a problem of drug distribution, which is a central notion in the emerging field of transport oncophysics^1,2^. Namely, the ability to exclusively direct and trap therapeutic agents in diseased tissues would enable the use of curative drug concentrations. Consequently, there is an urgent need to develop strategies for obtaining localized delivery of systemically administered therapeutics.

A promising method for improving drug distribution is the use of nanocarriers, which display favorable transport properties in the circulatory system, including reduced renal clearance and the enhanced permeability and retention (EPR) effect^3^. Currently there are several clinically approved nanodrugs on the market and many more are undergoing preclinical investigation^4,5^. Although nanodelivery generally results in a 10 to 100-fold improvement in tumor accumulation compared to conventional drugs^6,7^, a report summarizing the findings from 117 nanoparticle biodistribution studies found that less than 1% of the injected dose typically reaches the tumor^8^. Up to 99% of the dose deposits in the liver and spleen, which contain resident macrophages that form the mononuclear phagocyte system (MPS)^9–11^. These macrophages recognize and engulf foreign material, making the innate immune system the major barrier for delivery of nanocarriers to tumors. Accordingly, a variety of nanoparticle design approaches have been utilized to reduce or delay clearance by the MPS, the most notable being pegylation^12^. However, nanoparticle functionalization strategies aimed at decreasing macrophage interactions have resulted in marginal improvements in biodistribution^9,13^. Less emphasis has been placed on modulating the microenvironment in the liver and spleen to reduce nanoparticle uptake^14^. Therefore, it is of critical importance to explore the potential of MPS preconditioning strategies for improved drug delivery. Notably, such strategies should focus on transient deactivation of macrophages, as complete elimination of these cells has been shown to be toxic and even fatal in animal studies^15^.

Here, we propose a pretreatment strategy for temporary reduction of macrophage activity in the MPS through pharmacological inhibition of endocytosis. Specifically, the clinically approved antimalarial drug chloroquine was identified as an effective inhibitor of macrophage-specific nanoparticle uptake. The performance of chloroquine as a novel macrophage-preconditioning agent for improved biodistribution of soft and hard nanoparticles was evaluated in mouse models. The priming of resident macrophages with a clinically relevant dose of chloroquine decreased nanoparticle accumulation in the mononuclear phagocyte system and improved intratumoral delivery. Moreover, pretreatment with chloroquine increased the organotropic accumulation of particles designed to target the lungs. Taken together, chloroquine-induced modulation of innate immunity could serve as a broadly applicable and simple approach for improving nanodelivery.

## Results

### Nanoparticle uptake in macrophages

**Figure 1:**
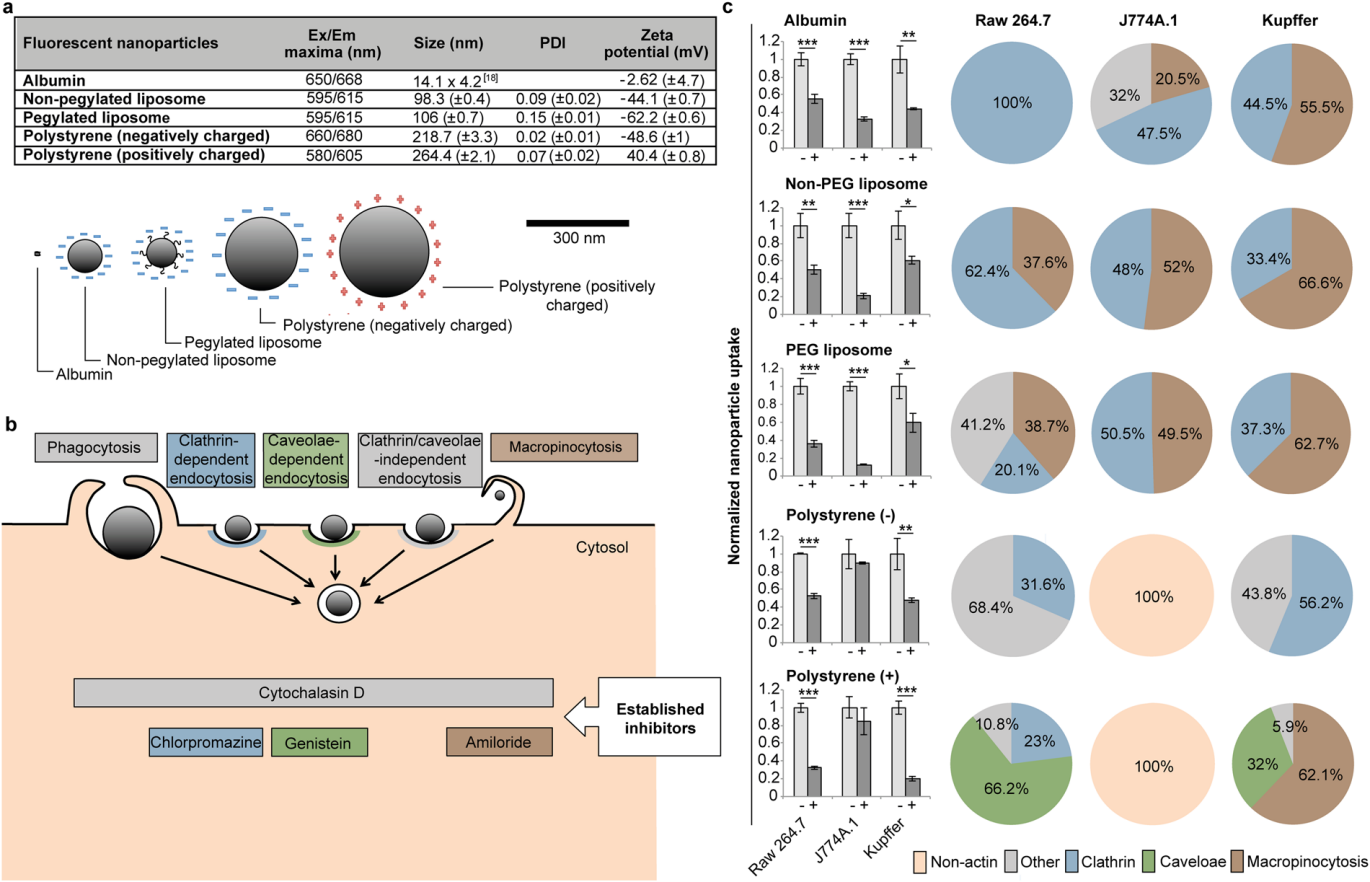
Nanoparticle characterization and uptake in macrophages. (**a**) Characterization of nanoparticles. (**b**) Schematic illustration of the various pathways of nanoparticle uptake in macrophages. Inhibitors of specific pathways are shown. Cytochalasin D was used as a broad-spectrum inhibitor of actin-dependent uptake. (**c**) Nanoparticle uptake in macrophages. Suppression of nanoparticle uptake upon exposure to cytochalasin D (bar graph ‘+’) in Raw 264.7, J774A.1, and Kupffer cells. Values are normalized to those of control cells. Data is presented as mean ± s.d. of triplicates. Statistics by Student’s *t*-test. *P < 0.05; **P < 0.01; ***P < 0.001, compared with control cells. Predominant pathways of nanoparticle uptake in the macrophage cell lines (pie chart). Values are normalized to those of cells treated with cytochalasin D. Other pathways of uptake refer to phagocytosis or clathrin/caveolae-independent endocytosis. PDI, polydispersity index; PEG, polyethylene glycol.

In order to develop strategies for reducing nanoparticle uptake by macrophages it is critical to understand the uptake mechanism. Although size and charge-dependent effects on nanoparticle uptake have previously been investigated, most studies have focused on nanoparticle internalization in non-phagocytic cells and in a subset of macrophages^16,17^. Notably, there are limited studies on the pathways of nanoparticle uptake in resident macrophages such as Kupffer cells. Here, a fluorescence assay for rapid evaluation of nanoparticle uptake was used to investigate internalization pathways in J774A.1 macrophage cells, Raw 264.7 macrophage cells, and immortalized Kupffer cells. Nanoparticles with a wide range of properties and sizes were assessed (Fig. 1a). Specifically, albumin, with a size of 14.1 nm × 4.2 nm^18^, was chosen as an example of a naturally occurring nanoparticle. The uptake of non-pegylated and pegylated liposomes (~100 nm) was also assessed, as they represent one of the largest categories of approved nanoparticles on the market^19^. Additionally, polystyrene nanoparticles (~240 nm) were used to evaluate charge-mediated effects on cellular uptake. Cells were visualized with fluorescence microscopy to confirm that washing steps were successful and that nanoparticle internalization had occurred (Supplementary Fig. 1). Specific pathways of particle uptake were suppressed using well-established small molecule inhibitors^20,21^ (Fig. 1b). Specifically, cytochalasin D was used as a broad-spectrum inhibitor of actin-dependent uptake. This inhibitor has previously been demonstrated to block phagocytosis, macropinocytosis, clathrin-dependent endocytosis, and caveolae-dependent endocytosis^22–24^. Additionally, chlorpromazine, genistein, and amiloride were used to suppress clathrin-mediated endocytosis, caveolae-mediated endocytosis, and macropinocytosis, respectively. Cell viability measurements were performed to ensure that inhibitors did not cause cell death during the internalization study (Supplementary Fig. 2). The results reveal that cytochalasin D pretreatment decreases cellular uptake of all nanoparticles, except for polystyrene particles in J774A.1 cells (Fig. 1c). Notably, the other inhibitors also failed to block the uptake of polystyrene particles in this cell line. Indeed, studies have shown that nanoparticle endocytosis can in certain instances take place in the absence of actin assembly^25^. The most common pathways of nanoparticle uptake in the macrophage cell lines studied were macropinocytosis and clathrin-mediated endocytosis (Fig. 1c). Interestingly, pegylated and non-pegyalted liposomes were internalized by similar means. The only nanoparticles that were engulfed through caveolae-mediated endocytosis were positively charged polystyrene particles. Although some common trends of uptake can be observed from these studies, the uptake heterogeneity across cell lines and nanoparticles, indicates that a complex set of factors govern macrophage and nanoparticle interactions.

### Pharmacological inhibition of nanoparticle uptake

**Figure 2:**
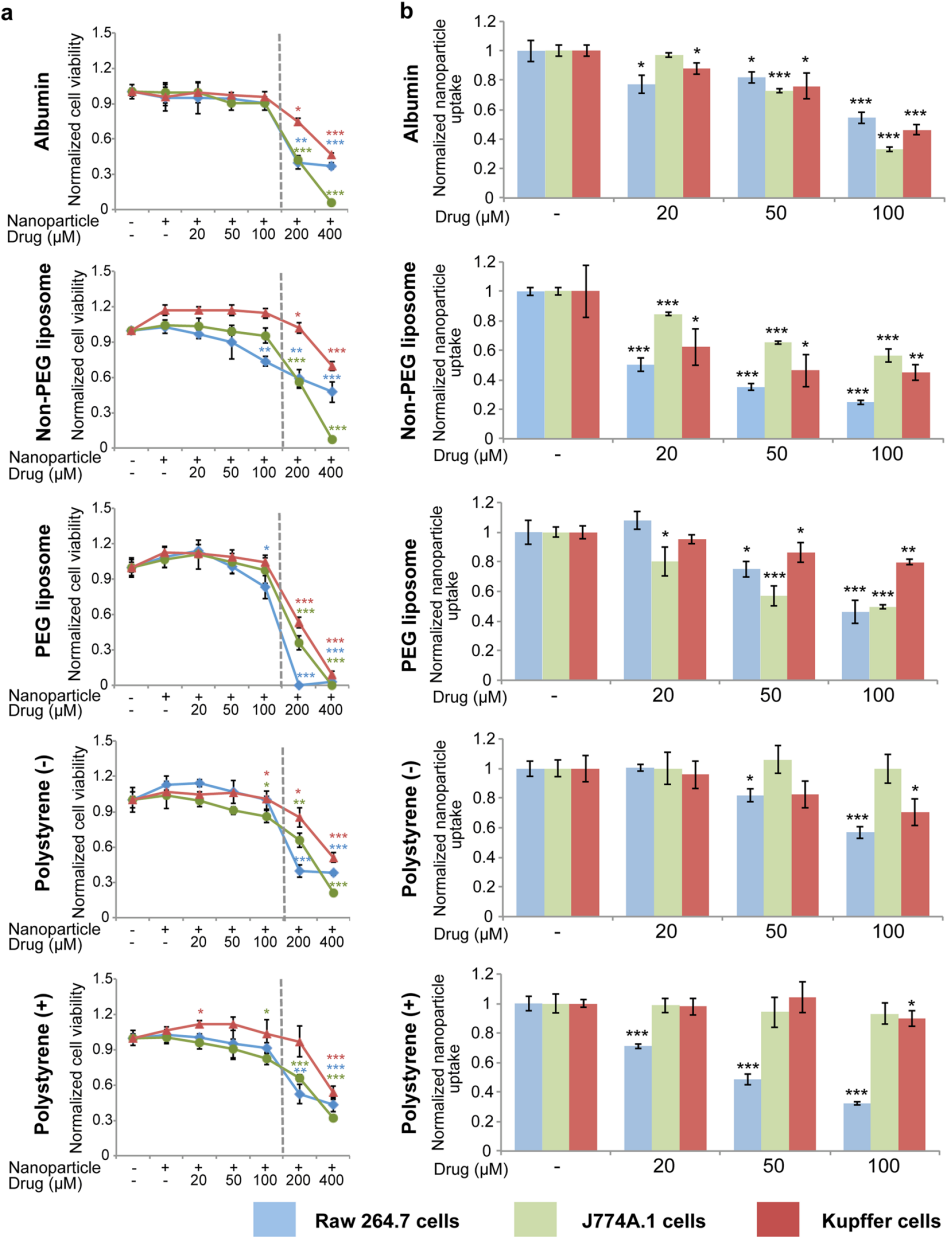
Effect of chloroquine on nanoparticle uptake in macrophages. (**a**) Viability of Raw 264.7, J774A.1, and Kupffer cells in response to chloroquine and nanoparticles. The left side of the dashed line indicates drug concentrations used in the nanoparticle uptake study. (**b**) Suppression of nanoparticle uptake upon exposure to chloroquine in Raw 264.7, J774A.1, and Kupffer cells. Values are normalized to those of control cells. Data is presented as mean ± s.d. of triplicates. Statistics by Student’s *t*-test. *P < 0.05; **P < 0.01; ***P < 0.001, compared with cells exposed to nanoparticles.

The cell-based assay that was utilized for assessment of internalization pathways also provided a rapid screening approach for identification of pharmacological inhibitors of nanoparticle uptake. The aim of the study was to develop a drug repositioning strategy for suppression of nanoparticle uptake by the mononuclear phagocyte system. An initial literature review identified the following four clinically approved drugs that have previously been shown to affect particle uptake by cells: i) pimozide, a United States Food and Drug Administration (FDA)-approved antipsychotic drug, ii) chloroquine, a FDA-approved antimalarial agent, iii) rapamycin, a FDA-approved immunosuppressive drug, and iv) Fasudil, a vasodilator approved in Japan. Specifically, pimozide was previously shown to decrease the uptake of bacteria in bone marrow-derived macrophages^26^, while chloroquine caused reduced internalization of dextran in retinal pigment epithelial cells^27^. Additionally, rapamycin was reported to reduce the uptake of albumin and dextran by dendritic cells^28^, while Rho-kinase inhibitors similar to Fasudil have been demonstrated to suppress the uptake of dextran in macrophages^29^. Prior to assessing nanoparticle uptake, cell viability assays were performed to identify drug doses that were non-toxic to macrophages (Fig. 2a, Supplementary Fig. 3a, and Supplementary Fig. 4a). Among the four drugs, chloroquine was the most effective at reducing particle uptake (Fig. 2b, Supplementary Fig. 3b, and Supplementary Fig. 4b). In particular, chloroquine had an effect on nanoparticle uptake even at lower doses (20 μM), while the other drugs were ineffective at this dose. The most dramatic effect of chloroquine was seen with non-pegylated liposomes. Chloroquine was selected as the agent of choice for further studies, as this drug displayed the highest potency as an uptake inhibitor and was previously shown to have a high volume of distribution and slow elimination *in vivo* ^30,31^. In rats, chloroquine was found to accumulate in high levels in the liver and spleen^30,32^. Specifically, a single oral dose of 40 mg/kg chloroquine resulted in a liver and spleen concentration of 58.5 mg/kg and 92.2 mg/kg, respectively^32^. These concentrations correspond roughly to a tissue concentration of 200 μM, which is higher than that used in the cell culture uptake studies.

**Figure 3:**
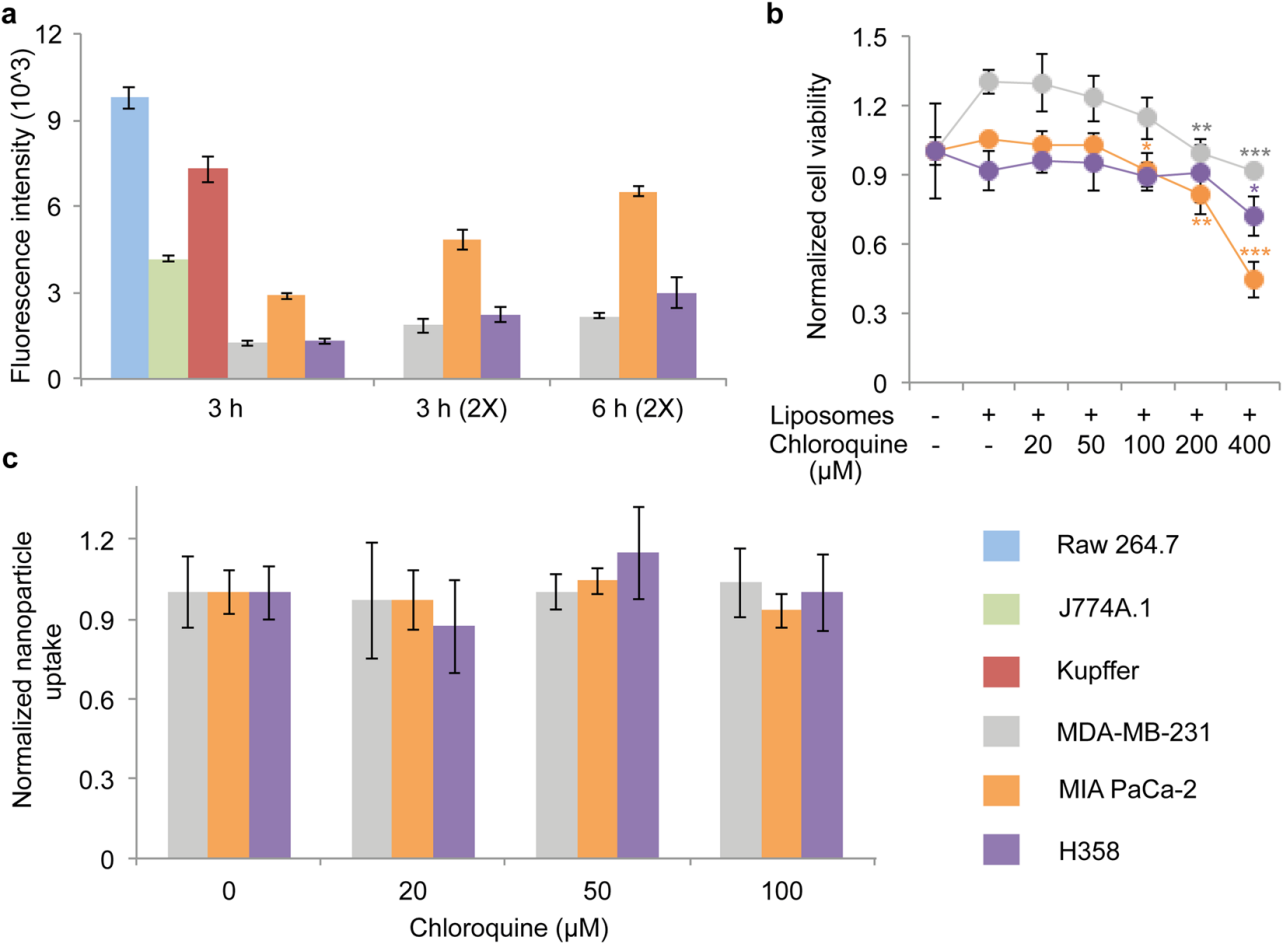
Effect of chloroquine on nanoparticle uptake in cancer cells. (**a**) Comparison of liposome (non-pegylated) uptake in macrophages (Raw 264.7, J774A.1, and Kupffer cells) and cancer cells (MDA-MB-231 breast cancer cells, MIA PaCa-2 pancreatic cancer cells, H358 lung cancer cells). (**b**) Viability of cancer cells in response to chloroquine and liposomes (6 h). (**c**) Effect of chloroquine on liposome uptake in MDA-MB-231 cells (6 h). Values are normalized to those of control cells. Data is presented as mean ± s.d. of triplicates. Statistics by Student’s *t*-test. **P < 0.01; ***P < 0.001, compared with cells exposed to liposomes.

Next, the effect of chloroquine on nanoparticle uptake in cancer cells was evaluated. The levels of nanoparticle uptake were substantially higher in J774A.1, Raw 264.7, and Kupffer cells compared to MDA-MB-231 breast cancer cells, MIA PaCa-2 pancreatic cancer cells, and H358 lung cancer cells. These results are expected, as macrophages are professional phagocytes designed to engulf foreign objects (Fig. 3a). Cancer cells were subjected to a two-fold increase in nanoparticle dose and incubation time in an attempt to obtain similar uptake levels as in the macrophages (Fig. 3a). To assess the effect of chloroquine on nanoparticle uptake in cancer cells, a non-toxic dose range was established (Fig. 3b). The results indicate that even at higher doses (100 μM), chloroquine does not reduce nanoparticle uptake in non-phagocytic cells, suggesting that chloroquine-mediated suppression of particle uptake may be macrophage-specific.

### Chloroquine-induced changes in Kupffer cells

**Figure 4:**
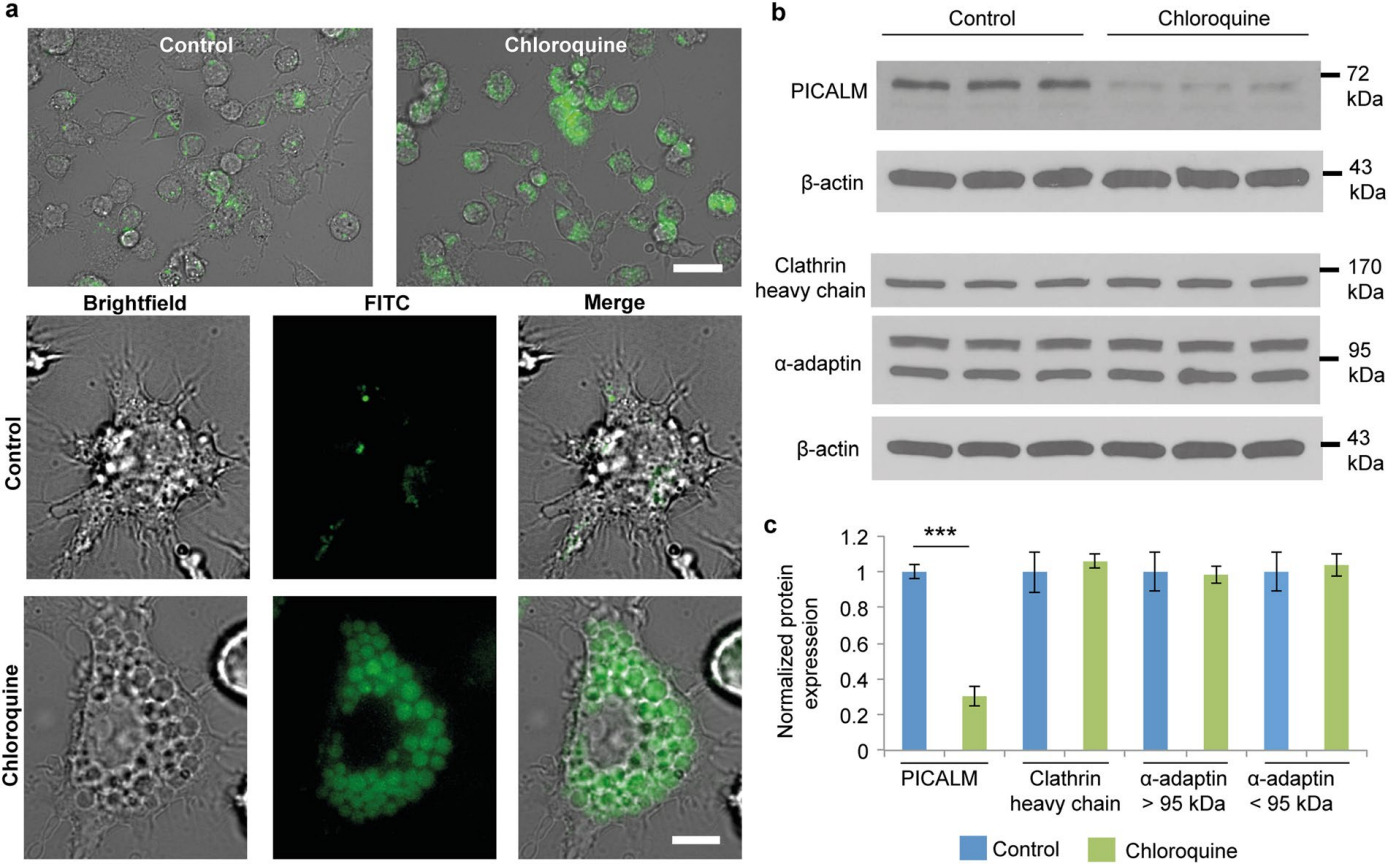
Chloroquine-induced changes in Kupffer cells. (**a**) Microscopy images of chloroquine-induced vacuole formation in live cells. Cells were pretreated with 100 μM of chloroquine. Lysotracker, green. Scale bar, 50 μm (upper), 10 μm (lower). (**b**) Western blot analysis of phosphatidylinositol-binding clathrin assembly protein (PICALM), α-adaptin, and clathrin heavy chain expression in cells. β-actin was used as a loading control. (**c**) Densitometric analysis of western blot results. Results represent the ratio between the protein of interest and β-actin (mean ± s.d. of three samples), and are normalized to control cells. Statistics by Student’s *t*-test. ***P < 0.001.

Chloroquine accumulates in the digestive vacuole of malaria-causing plasmodium parasites, where it interferes with hemoglobin degradation^33^. This lysosomotropic behavior is also evident in animal cells, where chloroquine increases lysosomal pH. Chloroquine has previously been shown to induce vacuole formation in cells due to lysosomal dysfunction^34–36^. In this study, confocal microscopy was performed to assess whether chloroquine induces vacuole formation in Kupffer cells. The results indicate that chloroquine caused widespread formation of acidic vacuoles (Fig. 4a), indicating that lysosomal function was disrupted. However, the cells recovered after removal of chloroquine from the cell culture media, demonstrating that chloroquine-induced lysosomal dysfunction is a reversible process (Supplementary Fig. 5a,b). The lysosome is usually the final compartment of the endocytosis process, which is the most common mechanism of nanoparticle internalization in cells^37^. Therefore, it can be expected that chloroquine would interfere with the later stages of endosomal trafficking of nanoparticles. However, apart from a study with dextran^27^, it has not previously been determined whether and to what extent chloroquine suppresses nanoparticle uptake in cells. Moreover, the mechanism by which chloroquine would affect earlier stages of nanoparticle endocytosis is currently unknown. Liquid chromatography tandem-mass spectrometry (LC-MS/MS) studies were performed to assess whether chloroquine induced changes in the levels of proteins involved in early stages of endocytosis. The study identified 25 and 19 proteins (≥2 unique peptides) that were exclusively detected in control cells and chloroquine-treated cells, respectively (Supplementary Fig. 6 and Supplementary Dataset 1). Many of these proteins were cytoskeletal or ribosomal proteins. Among the proteins exclusively detected in the control cells, phosphatidylinositol binding clathrin assembly protein (PICALM) stood out as it plays a critical role in clathrin-mediated endocytosis. In fact, studies have demonstrated that depletion of PICALM inhibits clathrin-dependent endocytosis^38^. Western blot analysis confirmed that chloroquine suppresses PICALM expression (Fig. 4b,c and Supplementary Fig. 7). In addition to PICALM, clathrin and AP2 are the most abundant proteins found in endocytic clathrin-coated vesicles^38^. The levels of AP2 and clathrin were unchanged in response to chloroquine (Fig. 4b,c), demonstrating that this drug specifically inhibits PICALM expression, as opposed to causing a general suppression of proteins involved in clathrin-mediated endocytosis. Taken together, these results shed light on a previously unknown mechanism by which chloroquine may prevent nanoparticle uptake in macrophages. However, it is worth noting that chloroquine was more effective than chlorpromazine (inhibitor of clathrin-dependent uptake) at blocking liposome uptake in Kupffer cells (Supplementary Fig. 8), suggesting that additional mechanisms of chloroquine-induced uptake inhibition may be involved.

### Chloroquine-induced changes in nanoparticle biodistribution

**Figure 5:**
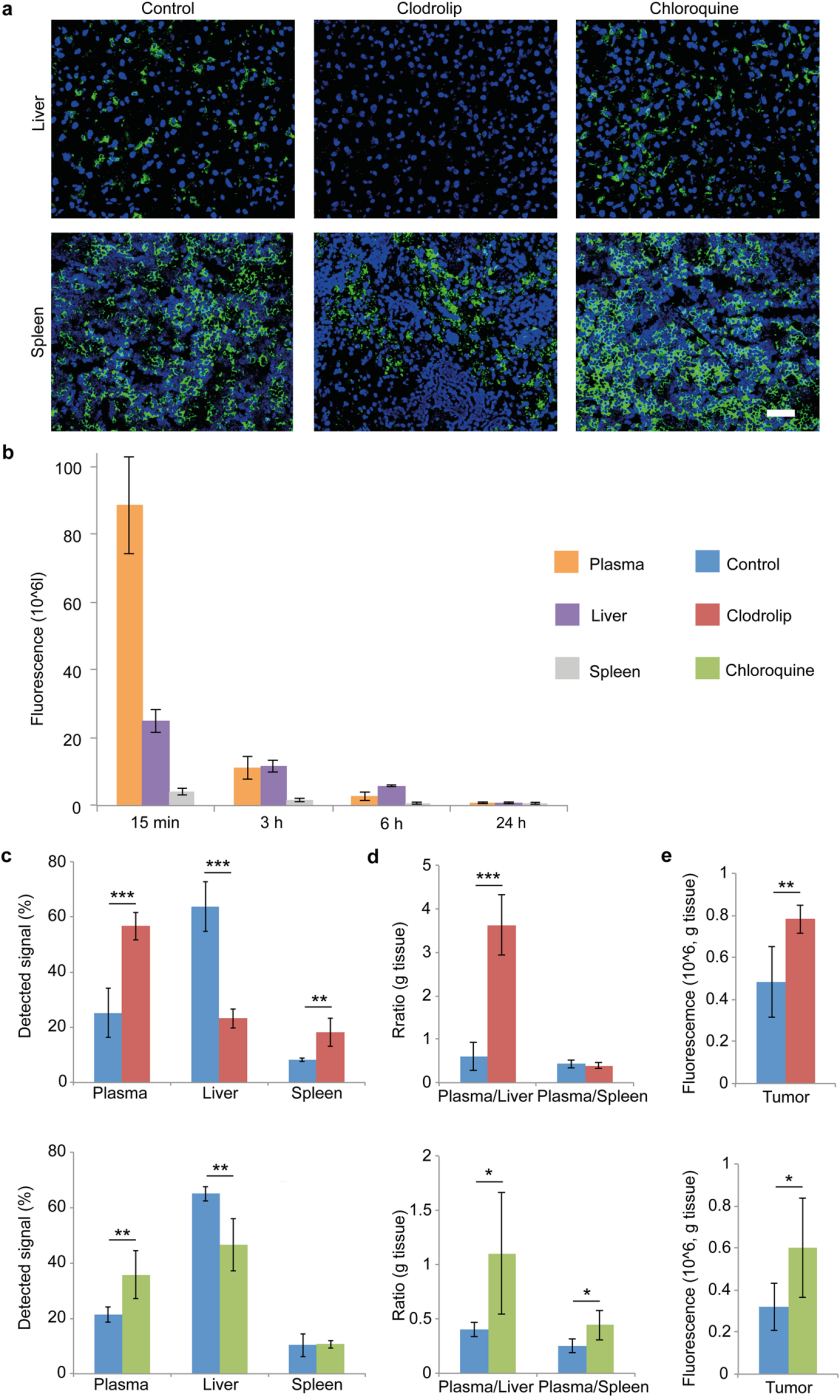
Effect of chloroquine on the biodistribution of liposomes. (**a**) Immunofluorescence staining of macrophages in the liver. Athymic nude mice were treated with clodronate liposomes (clodrolip; 50 mg/kg clodronate i.v.) or chloroquine (60 mg/kg/day i.p. for 7 days). DAPI, blue; macrophages (F4/80), green. Scale bar, 50 μm. (**b**) Accumulation of intravenously injected fluorescent liposomes in the plasma, liver, and spleen. The blood was collected and the liver and spleen were harvested 15 min, 3 h, 6 h, or 24 h post-injection of liposomes. (**c,d,e**) Effect of Kupffer cell depletion (clodrolip; 50 mg/kg clodronate) and chloroquine pretreatment (60 mg/kg/day for 7 days) on the biodistribution of fluorescent liposomes. The blood was collected and the organs were harvested 6 h post-injection of liposomes. **(c)** Detected signal in the plasma, liver, and spleen. (**d**) Plasma/liver and plasma/spleen accumulation ratio of liposomes (g tissue). (**e**) Accumulation of fluorescent liposomes in MDA-MB-231 orthotopic breast cancer tumors. Data is presented as mean ± s.d. (*n* = 5). Statistics by Student’s *t*-test. *P < 0.05; **P < 0.01; ***P < 0.001.

The macrophage depletion agent clodrolip was used as a positive control to evaluate the role of Kupffer cells on liposome biodistribution in mice. Clodrolip was given at a dose that has previously been shown to primarily deplete Kupffer cells^39^. Confocal microscopy confirmed that clodrolip completely depleted Kupffer cells in the liver, while there was a decrease in the abundance of macrophages in the spleen (Fig. 5a). A clinically relevant dose of chloroquine for mouse studies was determined by taking into account body surface area and bioavailability (see Methods section). The abundance of macrophages in the liver and spleen was unchanged in response to chloroquine treatment (Fig. 5a). Prior to assessing the effect of chloroquine and clodrolip on liposome biodistribution, the accumulation of intravenously injected fluorescent liposomes in the plasma, liver, and spleen at various time points was evaluated in control mice. Liposome accumulation in other organs was negligible (lungs: 1.1 ± 0.1%, heart: 0.4 ± 0.1%, kidneys: 1.6 ± 0.3% of the detected signal after 6 h). Stability studies demonstrated that the liposomes remained stable under physiological conditions (Supplementary Fig. 9), suggesting that the fluorescence detected in homogenized organs originated from the nanoparticles. The results revealed that liposome accumulation in the liver was higher than in the plasma after 6 h (Fig. 5b), making this time point ideal for studying inhibition of the mononuclear phagocyte system. Pretreatment with clodrolip or chloroquine decreased liver accumulation and increased the plasma concentration of liposomes (Fig. 5c), indicating that Kupffer cells are a major contributing factor to liver tropism of nanoparticles. Moreover, chloroquine increased the plasma/spleen liposome accumulation ratio, while this ratio remained unchanged in response to clodrolip (Fig. 5d). Although preconditioning with clodrolip led to a greater reduction in liver deposition of liposomes (64% decrease) compared to chloroquine pretreatment (28.5% decrease), complete depletion of Kupffer cells has been shown to cause adverse side effects and fatalities in mice^15^. Therefore, it is unlikely that this approach would be suitable for clinical use. On the contrary, chloroquine has been used in the clinic for malaria treatment since the 1940s. Hematoxylin and eosin (H&E) staining of the liver and spleen demonstrated that clodrolip caused gross morphological changes, while the chloroquine group was undistinguishable from the control group (Supplementary Fig. 10). Specifically, decreased cellularity was seen in the red pulp of the spleen in response to clodrolip treatment. The effect of clodrolip and chloroquine on liposome accumulation in tumors was evaluated in mice bearing MDA-MB-231 orthotopic breast cancer tumors. Both agents improved the tumoritropic delivery of liposomes (~2-fold) (Fig. 5e). Chloroquine did not affect body weight, tumor growth, or tumor weight during the pretreatment period (Supplementary Fig. 11).

**Figure 6:**
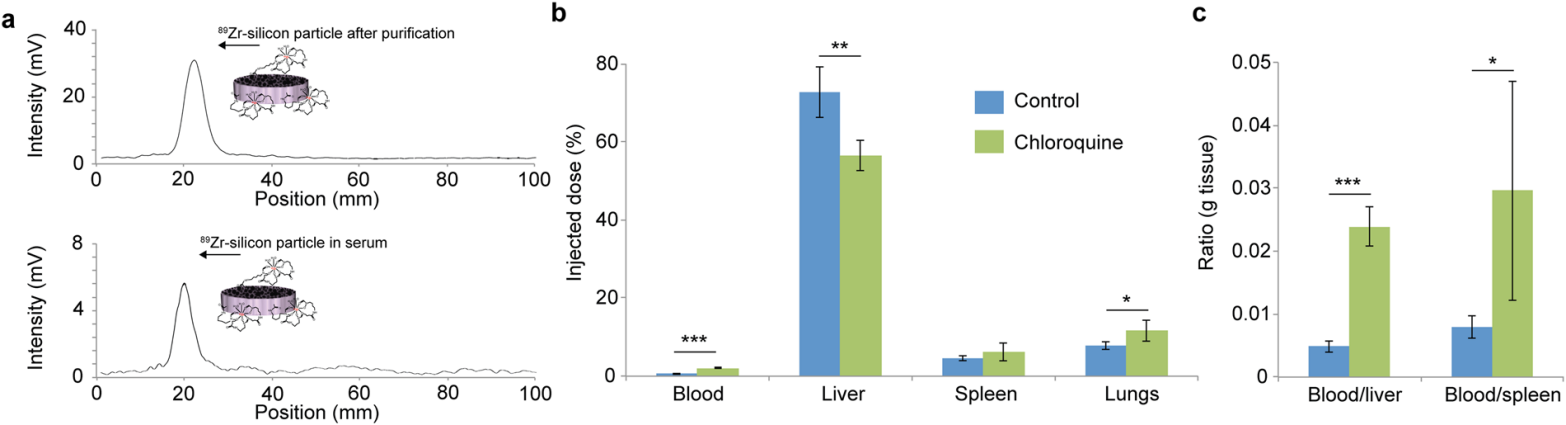
Effect of chloroquine on the biodistribution of silicon particles. **(a)** Stability of ^89^Zr-labeled silicon particles after 15 min in 50% mouse serum measured with a radio thin-layer chromatography (TLC) scanner. **(b)** Accumulation of intravenously injected ^89^Zr-labeled silicon particle in the blood, liver, spleen, and lungs. BALB/c mice were pretreated with chloroquine (60 mg/kg/day for 7 days). Organs were harvested 15 min after intravenous injection of silicon particles (8 × 10^7^/mouse). **(c)** Blood/liver and blood/spleen accumulation ratio of ^89^Zr-labeled silicon particles (g tissue). Data is presented as mean ± s.d. (*n* = 5). Statistics by Student’s t-test. *P < 0.05; **P < 0.01; ***P < 0.001.

In addition to increasing tumor accumulation, we investigated whether chloroquine could improve the delivery of particles designed to deposit in specific organs. For instance, discoidal silicon particles have been widely used in the preclinical setting and their size and shape can be tailored to obtain organotropic accumulation^40–46^. For instance disc-shaped silicon particles with a size of 700 nm × 2600 nm have been shown to deposit in lung tissue^47–49^. The stability of such radiolabeled silicon particles in serum was determined prior to assessing the effect of chloroquine pretreatment (Fig. 6a). Biodistribution studies were conducted after 15 min as 99.5% of the injected dose had already left the circulatory system at this time point (Fig. 6b). Pretreatment with chloroquine resulted in a 22% reduction in liver accumulation and a 3.9-fold increase in blood concentration of silicon particles (Fig. 6b). Moreover, this preconditioning strategy successfully increased lung accumulation from 7.8% to 11.7% of the injected dose (*P* < 0.5) (Fig. 6b). There was also a substantial increase in both the blood/liver and blood/spleen accumulation ratios (Fig. 6c). The impact of chloroquine on silicon particle deposition in other organs was also evaluated. Although chloroquine did not change the accumulation of silicon particles in muscle tissue and in the kidneys, there was an increase in heart accumulation (Supplementary Fig. 12). However, only 0.1% of the injected dose was present in the heart in chloroquine-treated mice. Previously, the uptake of discoidal silicon microparticles by macrophages has been studied *in vitro* ^50^. Here, cell culture uptake studies correlated with biodistribution results that demonstrated reduced liver accumulation of silicon particles in response to chloroquine pretreatment. Specifically, drug exposure caused a 58% reduction in particle uptake by Kupffer cells (Supplementary Fig. 13).

## Discussion

This study demonstrates the novel use of chloroquine as an MPS-preconditioning agent for improved site-specific delivery of particulate delivery systems. Notably, chloroquine treatment lead to a reduction in nanoparticle endocytosis by macrophages, while uptake levels in cancer cells remained unchanged in response to the drug. Moreover, in mice studies, chloroquine decreased liver and spleen accumulation of intravenously administered soft and hard nanomaterials. Chloroquine pretreatment also enhanced the tumor accumulation of non-pegylated liposomes and the site-specific localization of silicon particles in the lungs. Although considerable emphasis has been placed on nanoparticle design approaches and priming strategies targeted at the tumor microenvironment, preconditioning of healthy organs has been largely overlooked^14^. Accordingly, the most common approach for avoiding MPS clearance is conjugation of polyethylene glycol (PEG) to the surface of nanoparticles^12^. However, this strategy has resulted in marginal improvements in nanoparticle biodistribution^9,13^. Importantly, pegylation primarily delays MPS uptake rather than preventing the process. For example, in the case of lipid nanoparticles, PEG-phospholipids are prone to detachment from the surface after *in vivo* injection^51,52^, eventually making the nanoparticles susceptible to MPS clearance. Although, pegylation is a more effective strategy than macrophage depletion at preventing liver uptake after shorter time points^53^, this does not necessarily result in improved site-specific delivery of therapeutic agents. In fact, the PEG dilemma is a phrase coined to illustrate the lack of an MPS-specific response, i.e. pegylation reduces interactions with all types of cells, including cancer cells^54^. Paradoxically, pegylation can also lead to increased activation of the immune system, as illustrated by the accelerated blood clearance (ABC) phenomenon, in which nanoparticles are rapidly cleared from the circulation due to the formation of PEG-specific antibodies^13^. Additionally, in some cases, PEG can cause adverse reactions and accelerated nanoparticle clearance through activation of the complement system^55–57^. Taken together, there is a need for additional MPS-blocking strategies that can be used as an alternative or in combination with pegylation in order to improve drug delivery.

The promising results from this study encourage further development of microenvironmental priming strategies for modulation of innate immunity and improved drug delivery. It is worth noting that suppression of macrophage function may weaken the ability of the immune system to respond to pathogens and damaged cells. The foreseeable dosing regimen for MPS-preconditioning agents would be similar to that of nanotherapeutics, which are usually administered every 3–4 weeks^58,59^. Therefore, it is likely that the innate immune system would be able to recover in between treatments. In regards to chloroquine, the safety profile is well defined, as this drug has been used in patients for over 60 years^60^. In this study, a dose conversion from human to mouse studies was applied based on body surface area following FDA guidelines^61^. Mice received a chloroquine dose that was less than the standard human dose for malaria treatment. Notably, chloroquine is frequently administered on a continuous daily basis to patients with various autoimmune conditions^62^. The most common safety concern in these patients is damage to the eye due to chloroquine accumulation in the retinal pigment epithelium^62^.

Another important consideration for future implementation of this pretreatment approach is the effect of chloroquine on tumor-associated macrophages (TAMs). In fact, studies have demonstrated that TAMs can aid in the intratumoral uptake of nanoparticles in certain tumors^63–65^. Therefore, it may be necessary to tailor the chloroquine priming strategy to specific cancer types where TAMs do not play a critical role in tumor accumulation of nanoparticles. In addition to potentially affecting TAM-mediated drug delivery, chloroquine may also impact signaling between TAMs and other cells in the tumor microenvironment. In fact, macrophages generally play a tumor-promoting function through immunosuppression and stimulation of angiogenesis, cell survival, and invasion^65^. Therefore, chloroquine could potentially interfere with the ability of TAMs to support tumor progression. Notably, chloroquine has demonstrated anticancer activity in various animal models^66–68^ and is currently undergoing clinical investigation for the treatment of cancer^69^. In particular, chloroquine has been found to sensitize cancer cells to chemotherapy through the inhibition of authophagy^69^. Moreover, chloroquine has also been found to cause cancer vessel normalization^70^, which could potentially account for some of the improvement in intratumoral liposome accumulation observed in this study. It is worth emphasizing that regimens consisting of high doses of chloroquine in combination with chemotherapy for extended periods of time have demonstrated acceptable toxicity profiles in cancer patients^71^. Therefore, the combination of chloroquine with cancer nanotherapeutics is likely to be tolerable and have other advantages in addition to improving nanoparticle biodistribution.

## Methods

### Nanoparticles

Fluorescent polymeric nanoparticles (FluoroSpheres amine/carboxy-modified microspheres) and fluorescent albumin (albumin from bovine serum/alexa fluor 647) were purchased from Thermo Fisher Scientific. Fluorescent liposomes (dioleoyl-phosphatidylcholine/cholesterol liposomes labeled with Texas Red-dihexadecanoyl-phosphoethanolamine) were acquired from FormuMax Scientific. Discoidal porous silicon particles (2600 nm × 700 nm) were fabricated through photolithography and electrochemical etching of silicon wafers as previously described^43,72^. The silicon particles were modified with (3-aminopropyl)triethoxysilane (APTES; Sigma-Aldrich) as previously reported^43,72^. For fluorescent *in vitro* uptake studies, silicon particles were further conjugated to Alexa Fluor 647 NHS Ester (Life Technologies) according to a previously described protocol^73^. For radiolabeling, APTES-modified silicon particles were dispersed in anhydrous dimethylformamide (DMF; 5 × 10^9^ particles/mL; Sigma-Aldrich). Disuccinimidyl suberate (DSS; 2 mg/mL in DMF; 100 µL; Thermo Fisher Scientific) and triethylene amine (TEA; 20 µL; Sigma-Aldrich) were added to the solution and mixed for 30 min at room temperature. The solution was washed twice through centrifugation (1500 × g; 10 min) and mixed with deferoxamine (DFO) mesylate salt solution (3 mg/mL in dimethyl sulfoxide (DMSO); 100 µL; Sigma-Aldrich) and TEA-DMSO solution (1:45 v/v; 920 µL; Sigma-Aldrich) for 4 h at room temperature (30 sec sonication/h). The particles were then washed twice through centrifugation (1500 × g; 10 min) and then vacuum dried. DFO-modified particles were conjugated to ^89^Zr following a modified version of a previously reported procedure^74^. Briefly, Na_2_CO_3aq_ (1 M; 50–100 µL) was added to ^89^Zr-oxalate dispersed in oxalic acid (1 M) (from Washington University Medical School) and the pH was adjusted to 7.5–8. The DFO-modified silicon particles (1 × 10^9^ particles) were then added to the ^89^Zr solution (1–5 mCi), sonicated for 2 min, and mixed for 2 h at room temperature. The ^89^Zr -labeled silicon particles were washed through centrifugation and the purity was verified with an EZ-SCAN thin-layer chromatography (TLC) Scanner (Carroll & Ramsey Associates). For nanoparticle characterization, nanoparticles were diluted in 1:50 in distilled water. Nanoparticle size/polydispersity index (PDI) (dynamic light scattering) and zeta potential (laser Doppler micro-electrophoresis) were measured using a Zetasizer Nano ZS (ZEN 3600, Malvern Instruments) as previously described^75,76^. For each nanoparticle sample, five measurements were taken with 10 runs per measurement.

### Nanoparticle uptake and cell viability *in vitro*

Raw 264.7, J774A.1, MDA-MB-231, H358, and MIA PaCa-2 cells were acquired from ATCC, while immortalized rat Kupffer cells were purchased from Applied Biological Materials. Cells were propagated in Dulbecco’s modified Eagle’s Medium (DMEM) with 4.5 g/L glucose, L-glutamine, and sodium pyruvate (for Raw 264.7, J774A.1, and MDA-MB-231 cells; Corning), Prigrow II Medium (for Kupffer cells; Applied Biological Materials), or RPMI-1640 medium (for H358 cells; GE Healthcare Hyclone). The media was supplemented with 10% fetal bovine serum (FBS; Atlas Biologicals), 100 units/mL penicillin, and 100 μg/mL streptomycin (Sigma-Aldrich). Cells were maintained in a humidified incubator at 37 °C and 5% CO_2_. For nanoparticle uptake studies, FBS was replaced with species-specific serum (mouse serum from Abcam; rat and human serum from Sigma-Aldrich) to replicate *in vivo* conditions. Cells were grown to a confluency of 80% in black clear-bottom 96-well plates and pretreated for 30 min with chlorpromazine hydrochloride (30 μM; dissolved in water; Sigma-Aldrich), genistein (100 μM; dissolved in DMSO; Sigma-Aldrich), amiloride hydrochloride (500 μM for Raw 264.7 cells; 250 μM for 774 A.1 and Kupffer cells; dissolved in DMSO; Sigma-Aldrich), cytochalasin D (20 μM for Raw 264.7 cells; 10 μM for J774A.1 and Kupffer cells; dissolved in DMSO; MP Biomedical), chloroquine phosphate (20–400 μM; dissolved in water; Sigma-Aldrich), rapamycin (20–400 μM; dissolved in DMSO; Sigma-Aldrich), Fasudil dihydrochloride (20–400 μM; dissolved in water; Sigma-Aldrich), or pimozide (20–400 μM; dissolved in DMSO; Sigma-Aldrich). Nanoparticles were then added to the cells, consequently reducing the concentration of uptake inhibitors by half. Specifically, cells were exposed to fluorescent albumin (20 μg/mL), fluorescent non-pegylated liposomes (200 μM of lipids), fluorescent polystyrene particles (3 × 10^9^ particles/mL), or fluorescent silicon particles (6 × 10^7^ particles/mL) for 3 h. Alternatively, cells were subjected to pegylated liposomes (370 μM of lipids) for 6 h. Cancer cells were incubated with fluorescent non-pegylated liposomes (400 μM of lipids) for 6 h. After nanoparticle incubation, cells were washed three times with phosphate buffered saline (PBS; HyClone, Thermo Fisher Scientific) and fluorescence intensity was measured using a Synergy H4 Hybrid Microplate Reader (BioTek). The background fluorescence of untreated cells was subtracted from the obtained values. Fluorescent images of live cells were captured with an Eclipse Ti Inverted Fluorescence Microscope (Nikon). For visualization of acidic vacuoles, cells were treated with LysoTracker Green DND-26 (Life Technologies) according to the manufacturer’s instructions. Cells were then incubated with complete cell culture medium and cell viability was measured using the CellTiter AQueous one solution cell proliferation assay (Promega) according to the manufacturer’s instructions. The contribution of each cellular uptake pathway to nanoparticle internalization was plotted based on reduced fluorescence intensity compared to untreated cells. Values were further normalized to the reduced fluorescence intensity caused by cytochalasin D, a broad-spectrum inhibitor of actin-dependent uptake.

### LC-MS/MS

Kupffer cells (80% confluency) were treated with 100 μM of choroquine for 3 h, after which the cells were washed in PBS and lysed in M-PER Mammalian Protein Extraction Reagent (Thermo Fisher Scientific). Protein samples were obtained through centrifugation (21,000 × g; 10 min) and quantification was performed with a Pierce BCA Protein Assay Kit (Thermo Fisher Scientific). To reduce proteins, the samples were incubated in 25 mM NH_4_HCO_3_ buffer supplemented with freshly prepared 25 mM dithiothreitol for 20 min at 56 °C. Protein alkylation was then carried out in 25 mM NH_4_HCO_3_/50 mM iodoacetamide buffer in the dark for 20 min at room temperature. Protein digestion was performed in trypsin solution overnight at 37 °C. The tryptic peptides (10 μL) were injected into a nano-LC UltiMate 3000 high-performance liquid chromatography coupled with a LTQ Velos Pro LC-MS/MS system (Thermo Fisher Scientific). Peptides were separated by EASY-Spray C18 LC Columns (15 cm length × 75 μm internal diameter; 3 μm particle size; Thermo Fisher Scientific) with a 120 min linear gradient of 5–40% acetonitrile/0.1% formic acid using a 0.3 μL/min column flow rate. Peptides were analyzed with an LTQ Velos Pro mass spectrometer (Thermo Fisher Scientific) using a 3.0 kV electrospray source. For data-dependent acquisition, full MS scans were acquired over the mass-to-charge (m/z) ratio range 200–2000. The ten most intense peaks were selected for sequencing and fragmentation with 30% collision energy. Database searches were carried out in Proteome Discoverer 2.0 (Thermo Fisher Scientific) and Mascot was used to search the data against the Swissprot_Rattus database. The precursor and fragment mass tolerances were set to 15 ppm and 0.5 Da, respectively, with up to two missed cleavages. A signal-to-noise ratio threshold of 1.5 was applied.

### Western blot

Kupffer cells (80% confluency) were treated with 100 μM of choroquine for 3 h, after which the cells were washed in PBS and lysed in M-PER Mammalian Protein Extraction Reagent (Thermo Fisher Scientific) with Halt protease inhibitors (Thermo Fisher Scientific). Protein samples were obtained through centrifugation (21,000 × g; 10 min) and quantification was performed with a Pierce BCA Protein Assay Kit (Thermo Fisher Scientific). After electrophoresis on Mini-PROTEAN TGX precast gels (Bio-Rad), proteins were transferred to an Amersham Protran 0.1 μm nitrocellulose blotting membrane (GE Healthcare). Membranes were blocked in 10% milk and incubated with a clathrin heavy chain antibody (1:1000 dilution; Cell signaling, #2410), an alpha adaptin antibody (1:1000 dilution; Thermo Fisher Scientific, #MA3-061), a PICALM antibody (3:2000 dilution; Sigma-Aldrich, #SAB3500400), or a beta actin antibody (1:10000 dilution; Thermo Fisher Scientific, #MA1-91399) overnight at 4 °C. Membranes were then incubated with an anti-rabbit immunoglobulin G (IgG) horseradish peroxidase (HRP)-linked antibody (1:1000 dilution; Cell Signaling, #7074P2) or an anti-mouse IgG HRP-linked antibody (1:15000; Thermo Fisher Scientific; #31450) for 1 h at room temperature. Protein bands were detected using a Pierce ELC Western Blotting Substrate (Thermo Fisher Scientific) and visualized on autoradiography films (Denville Scientific) using a Konica SRX 101a Film Processor (Konica Minolta). Densitometry measurements of protein bands were performed in ImageJ.

### Particle stability

Liposomes (25 μL) were incubated in Prigrow II Medium containing 10% FBS (0.9 mL) on a shaker at 37 °C. At various time points, 20 μL of the solution was removed and mixed with distilled water (980 μL). The size and PDI of the liposomes was determined by DLS as described in the nanoparticle section. For fluorophore detachment studies, liposomes (3 μL) were incubated in Prigrow II Medium containing 10% FBS (1.4 mL) on a shaker at 37 °C. Centrifugation (4,000 × g; 30 min) of the solution in Amicon Ultra-15 Centrifugal Filter Device 100K (Millipore Sigma) was performed to separate liposomes from detached fluorophores. Serial dilutions of the ultrafiltrate were placed in a black clear-bottom 96-well plate and assayed for fluorescence levels using a Synergy H4 Hybrid Microplate Reader (BioTek). The stability of ^89^Zr-labeled silicon particles (351 KBq/100 μL) was evaluated in saline containing 50% mouse serum (1 mL; 37 °C) using an EZ-SCAN TLC Scanner (Carroll & Ramsey Associates).

### Biodistribution studies

Animal studies were performed according to a protocol approved by the Animal Care and Use Committee at the Houston Methodist Research Institute and in adherence to the National Institutes of Health Guide for the Care and Use of Laboratory Animals. Female athymic nude mice (6–8 weeks of age; liposome study) and BALB/c mice (4–8 months of age; silicon particle study) were purchased from Charles River.

For liposome biodistribution studies, mice were intravenously injected with fluorescent non-pegylated liposomes (100 μL/mouse). At various time points, mice were euthanized for blood and organ collection (*n* = 5 for each time point). Blood was collected through cardiac puncture using needles pre-rinsed in EDTA (0.5 M; pH 8; Thermo Scientific). Plasma was obtained through centrifugation (10 min; 3,000 × g) of blood in Microtainer Tubes with K2E (BD). Preweighed organs were homogenized in PBS (1 g/3 mL) using a T25 Digital Ultra Turrax Homogenizer (Ika). Serial dilutions of the plasma and homogenized organs were placed in black clear-bottom 96-well plates and assayed for fluorescence levels using a Synergy H4 Hybrid Microplate Reader (BioTek). The background fluorescence of plasma and organ samples from untreated mice was subtracted from the obtained values to account for autofluorescence. To assess the effect of macrophage depletion on liposome biodistribution, mice received intravenous injections of PBS or clodronate liposomes (clodrolip; 50 mg/kg clodronate; Encapsula Nanosciences) 24 h prior to intravenous injections of fluorescent liposomes (100 μL/mouse; 50 mM lipids). For the chloroquine group, a dose translation from human to animal studies was performed based on body surface area^61^. In humans, the chloroquine phosphate dose for malaria treatments is 2.5 g taken orally over 3 days^77^. The bioavailability of chloroquine phosphate tablets in humans (89%)^78^ was also considered to account for the intraperitoneal administration route used in the mouse studies. The dose translations calculations yielded a total mouse dose of 457 mg/kg. A total dose of 420 mg/kg chloroquine phosphate spread out over 7 days (60 mg/kg/day; dissolved in water; intraperitoneal injection) was used in the biodistribution studies. Mice were intravenously injected with fluorescent liposomes (100 μL/mouse; 50 mM lipids) 1 h after the last chloroquine injection. Control, clodrolip-treated, and chloroquine-treated mice were euthanized 6 h after liposome injection and liposome biodistribution was assessed as described above (*n* = 5 for each group). For tumor studies, MDA-MB-231 breast cancer cells suspended in 50% phenol red-free matrigel matrix (BD) in PBS were injected into the mammary fat pad of athymic nude mice (3 × 10^6^ cells/mouse). Tumor volume was measured with the following formula: 0.5 × width^2^ × length. When the tumor volume reached ~100–200 mm^3^, tumor accumulation studies were initiated as described above.

For the silicon particle biodistribution study, mice were pretreated with chloroquine as described above. Control and chloroquine-treated mice were then intravenously injected with ^89^Zr-labeled silicon particles (8 × 10^7^/mouse) and euthanized after 15 min (*n* = 5 for each group). The activity of organs and blood was measured in counts per minutes (cpm) with a 2470 Wizard Automatic Gamma Counter (Perkin Elmer) and corrected for background and decay.

### Immunofluorescence and H&E staining

Athymic nude mice were treated with PBS, clodorlip, and chloroquine as described in the biodistribution section. Mice were sacrificed 24 h after clodrolip administration and 1 h after the last chloroquine injection. For immunofluorescence staining, liver and spleen sections were emerged in Tissue-Tek optimum cutting temperature (O.C.T) compound (VWR) on dry ice and stored in −80 °C. For H&E staining, liver and spleen sections were incubated in 10% buffered formalin phosphate (Thermo Fisher Scientific) and embedded in paraffin. For H&E staining, 6 μm tissue sections were stained with Leica Autostainer XL and images were captured using a Nikon Eclipse 80i. For immunofluorescence staining, 6 μm tissue sections were fixed in acetone, blocked in 2.5% normal horse serum (Vector Laboratories), and incubated with a F4/80 antibody (1:100 dilution; Bio-Rad, #MCA497A488) at 4 °C overnight. The slides were then incubated with an anti-rat IgG-Alexa Fluor-488 (1:200 dilution; Thermo Fisher Scientific, #A21208) for 1 h at room temperature. The slides were mounted with Vectashield Antifade Mounting Medium with DAPI (Vector Laboratories) and imaged on a Nikon A1 Confocal Imaging System.

### Statistical analysis

*T*-test comparisons (two-tailed, unpaired) were performed to evaluate statistical significance.

### Data availability

All data are available within the paper and its Supplementary Information files.

## Electronic supplementary material

Supplementary Information

Supplementary Dataset 1
