# Supplementary material for: A chloroquine-induced macrophage-preconditioning strategy for improved nanodelivery
Source: Sci Rep. 2017 Oct 23;7:13738. doi: 10.1038/s41598-017-14221-2 (PMC5653759; doi:10.1038/s41598-017-14221-2)
Supplement: Supplementary file 1 — Supplementary Information [file 41598_2017_14221_MOESM1_ESM.pdf]

SUPPLEMENTARY INFORMATION

A chloroquine-induced macrophage-preconditioning strategy for improved nanodelivery

Joy Wolfram[1,2,*], Sara Nizzero[1,3], Haoran Liu[1], Feng Li[1], Guodong Zhang[1], Zheng Li[1], Haifa Shen[1,4], Elvin Blanco[1], Mauro Ferrari[1,5,*]

[1]*Department of Nanomedicine, Houston Methodist Research Institute, Houston, TX 77030, USA.*
[2]*Department of Transplantation, Mayo Clinic, Jacksonville, FL 32224, USA.*
[3]*Applied Physics Graduate Program, Rice University, Houston, TX 77005, USA.*
[4]*Department of Cell and Developmental Biology, Weill Cornell Medicine, New York, NY 10065, USA.*
[5]*Department of Medicine, Weill Cornell Medicine, Weill Cornell Medicine, New York, NY 10065, USA.*
[*]*Corresponding authors.*

Supplementary figures

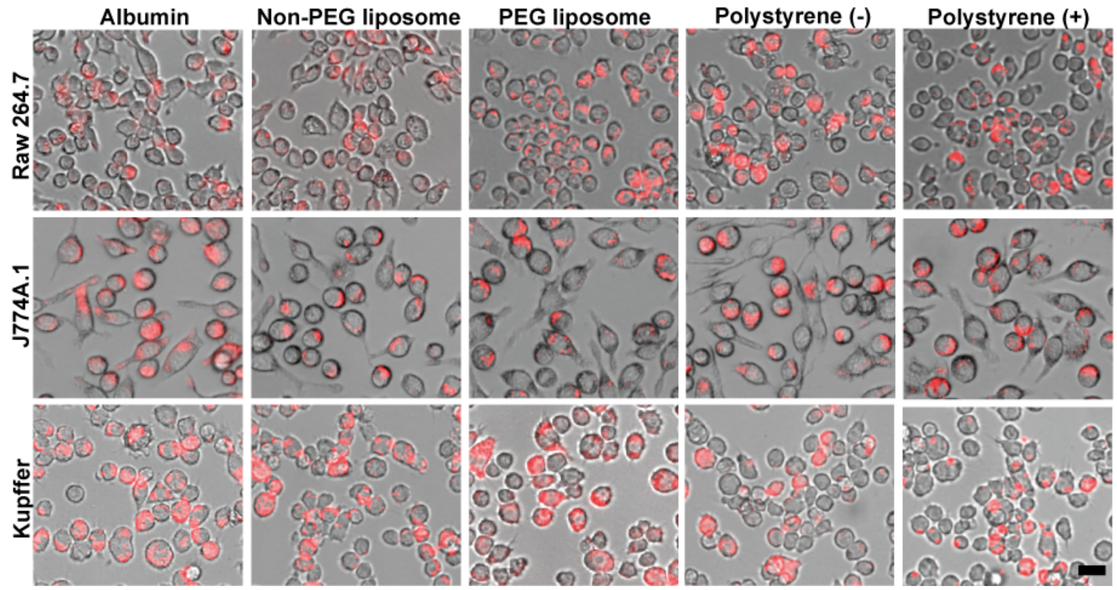

Supplementary Figure 1 | Nanoparticle uptake in macrophages. Fluorescence microscopy images of nanoparticle uptake in Raw 264.7 cells, J774A.1 cells, and Kupffer cells. Scale bar, 20 μm.

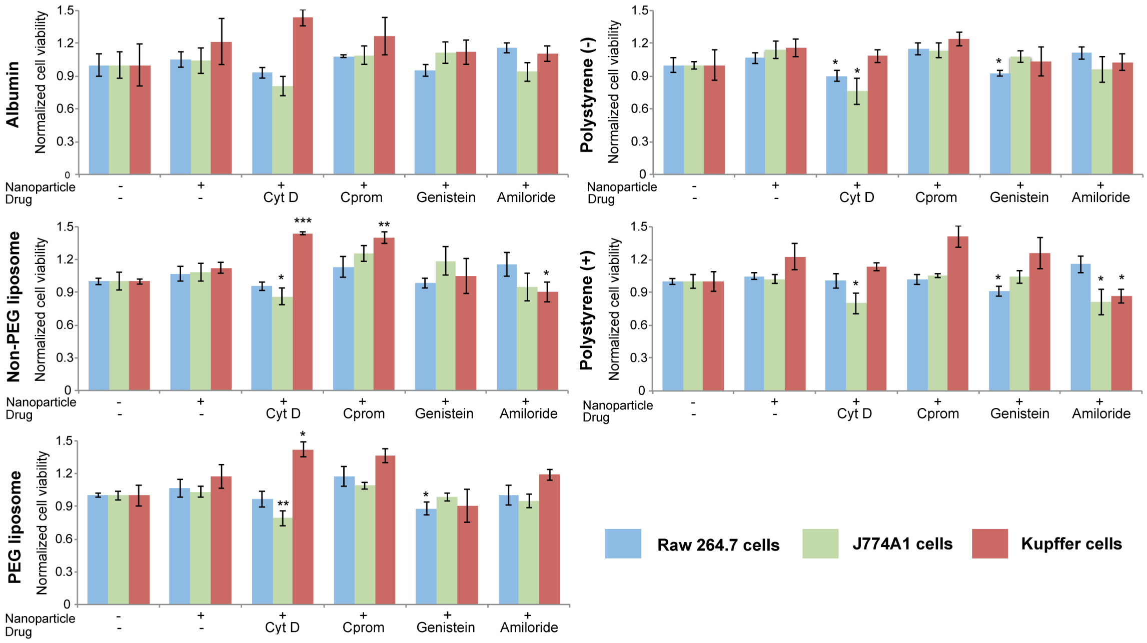

Supplementary Figure 2 | Viability of macrophages in response to nanoparticles and uptake inhibitors. Raw 264.7, J774A.1 and Kupffer cells were pretreated with inhibitors (30 min) prior to nanoparticle exposure (3 h or 6 h). Values are normalized to those of control cells. Data is presented as mean ± s.d. of triplicates. Statistics by Student's *t*-test. *, $P < 0.05$; **, $P < 0.01$; ***, $P < 0.001$, compared with cells exposed to nanoparticles. Cprom, chlorpromazine; CytD, cytochalasin D.

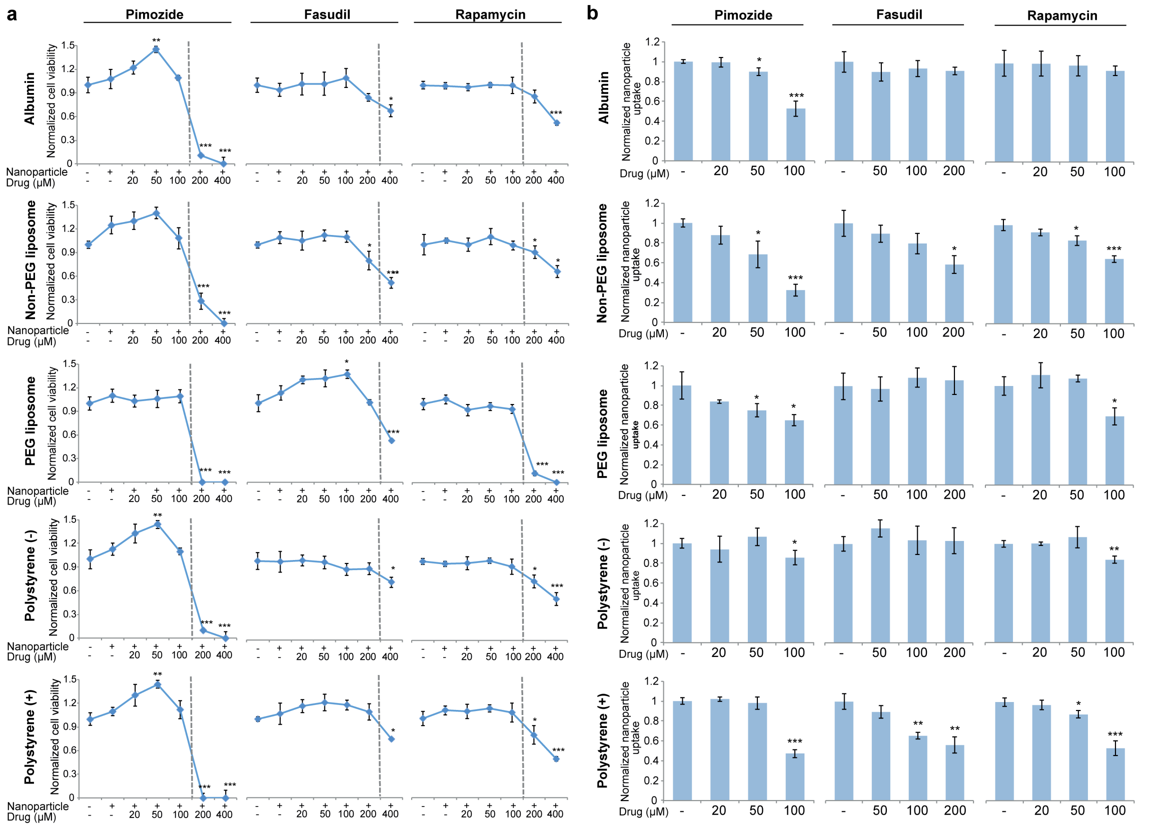

Supplementary Figure 3 | Effect of pimozide, Fasudil, and rapamycin on nanoparticle uptake in Raw 264.7 cells. (**a**) Cell viability upon exposure to drugs and nanoparticles. The left side of the dashed line indicates drug concentrations used in the nanoparticle uptake study. (**b**) Effect of drugs on nanoparticles uptake in cells. Values are normalized to those of control cells. Data is presented as mean ± s.d. of triplicates. Statistics by Student's *t*-test. *, $P < 0.05$; **, $P < 0.01$; ***, $P < 0.001$, compared with cells exposed to nanoparticles.

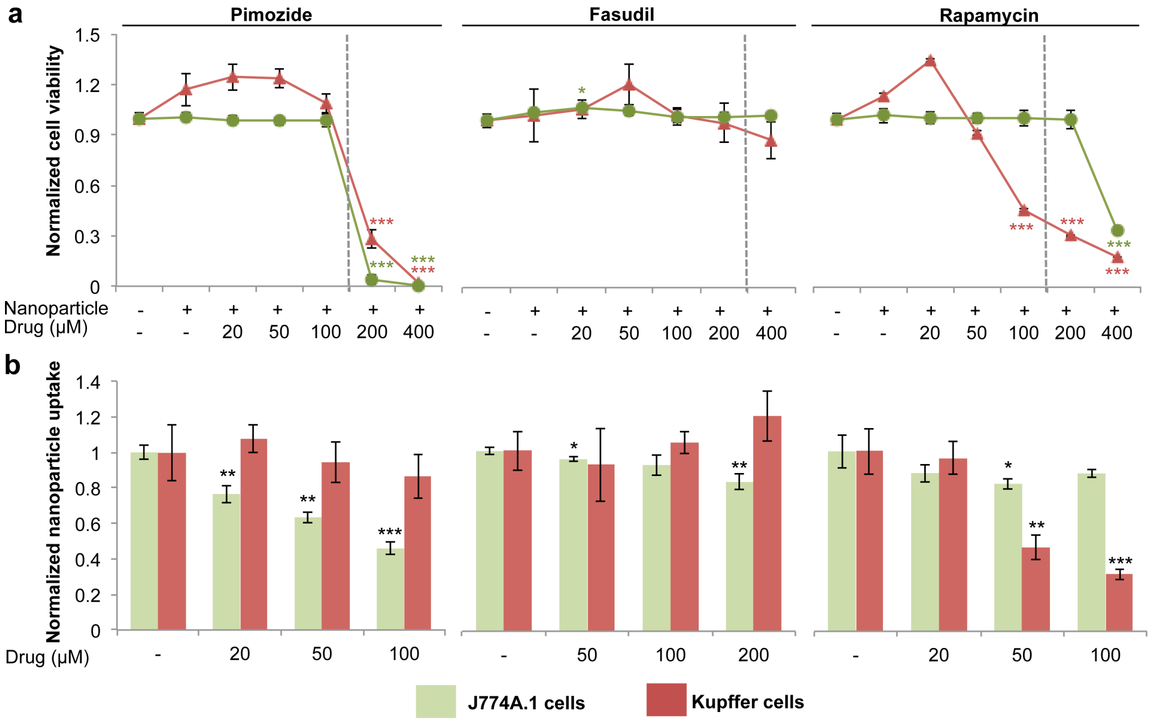

Supplementary Figure 4 | Effect of pimozide, Fasudil, and rapamycin on liposome uptake in J774A.1 and Kupffer cells. (**a**) Cell viability upon exposure to drugs and liposomes. The left side of the dashed line indicates drug concentrations used in the liposome uptake study. (**b**) Effect of drugs on liposome uptake in cells. Values are normalized to those of control cells. Data is presented as mean ± s.d. of triplicates. Statistics by Student's *t*-test. *, $P < 0.05$; **, $P < 0.01$; ***, $P < 0.001$, compared with cells exposed to nanoparticles.

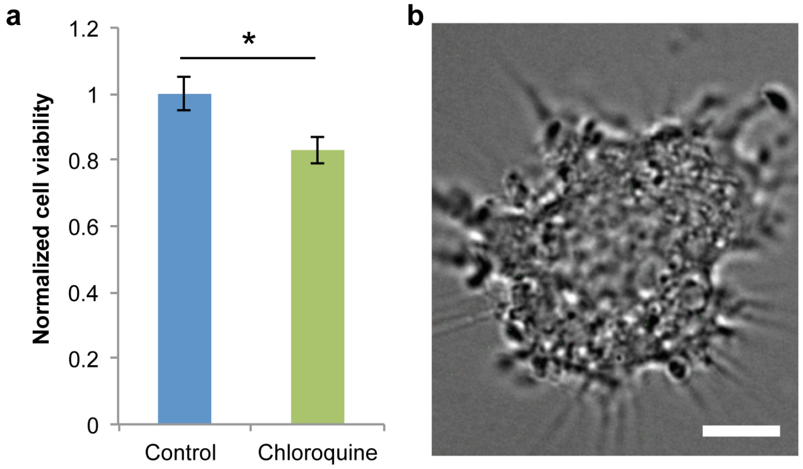

Supplementary Figure 5 | Ability of Kupffer cells to recover from chloroquine treatment. Kupffer cells were exposed to 100 µM chloroquine for 3 h, after which cells were incubated with fresh media for 24 h. (**a**) Cell viability. Values are normalized to those of control cells. Data is presented as mean ± s.d. ($n = 5$). Statistics by Student's *t*-test. *, $P < 0.05$. (**b**) Microscopy image of a live cell. Scale bar, 10 µm.

a

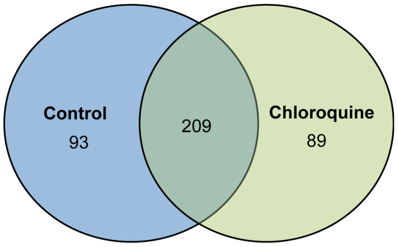

b

Control
Actin, gamma-enteric smooth muscle (ACTG2)
Tubulin beta-4B chain (TUBB4B)
Tubulin alpha-1A chain (TUBA1A)
EF-hand domain-containing protein D2 (EFHD2)
Adenosylhomocysteinase (AHCY)
60S ribosomal protein L5 (RPL5)
Leucine-rich repeat-containing protein 59 (LRRC59)
Actin-related protein (ACTR3)
Malignant T-cell-amplified sequence 1 (MCTS1)
Coatomer subunit beta' (COPB2)
60S ribosomal protein L3 (RPL3)
F-actin-capping protein subunit alpha-2 (CAPZA2)
Nuclear migration protein nudC (NUDC)
Eukaryotic translation initiation factor 3 subunit B (EIF3B)
Phosphatidylinositol-binding clathrin assembly protein (PICALM)
C-1-tetrahydrofolate synthase, cytoplasmic (MTHFD)
Translocon-associated protein subunit alpha (SSR1)
Nucleobindin-1 (NUCB1)
Nucleoside diphosphate kinase A (NME1)
40S ribosomal protein S3a (RPS3A)
Proteasome subunit alpha type-7 (PSMA7)
Coiled-coil domain-containing protein 47 (CCDC47)
Glucose-6-phosphate 1-dehydrogenase (G6PDX)
Renin receptor (ATP6AP2)
Phosphoglucomutase-1 (PGM1)

Chloroquine
Actin, aortic smooth muscle (ACTA2)
Tubulin alpha-1C chain (TUBA1C)
Cyclin-dependent kinase 1 (CDK1)
40S ribosomal protein S18 (RPS18)
Ras GTPase-activating protein 1 (RASA1)
Voltage-dependent anion-selective channel protein 1 (VDAC1)
Brain acid soluble protein 1 (BASP1)
Transformer-2 protein homolog beta (TRA2B)
Coatomer subunit gamma (COPG)
Nucleoside diphosphate kinase B (NME2)
Eukaryotic translation initiation factor 3 subunit C (EIF3C))
Dynamin-1-like protein (DNM1)
Fumarate hydratase, mitochondrial (FH)
Tyrosine-protein phosphatase non-receptor type 6 (PTPN6)
Isocitrate dehydrogenase [NAD] subunit alpha, mitochondrial (IDH3A)
Alpha-actinin-1 (ACTN1)
Proteasome subunit alpha type-1 (PSMA1)
Unconventional myosin-Ie (MYO1E)
28 kDa heat- and acid-stable phosphoprotein (PDAP1)

Supplementary Figure 6 | Protein expression in Kupffer cells in response to chloroquine. Protein lysates were analyzed with liquid chromatography tandem-mass spectrometry (LC-MS/MS) (**a**) Circles represent the amount of proteins expressed in each group. Proteins with a score ≥ 30 were considered. (**b**) Table of proteins exclusively detected in control cells and chloroquine-treated (100 μM) cells. Proteins with a score ≥ 30 and # of unique peptide ≥ 2 are presented.

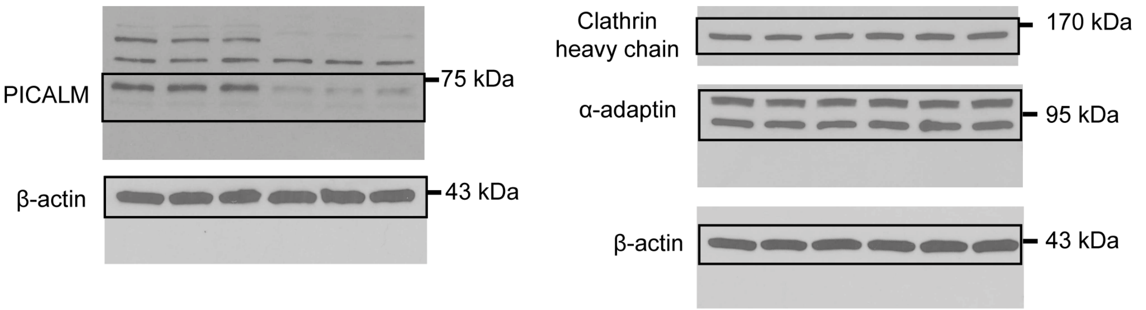

Supplementary Figure 7 | Uncropped Western blot images. PICALM, phosphatidylinositol-binding clathrin assembly protein.

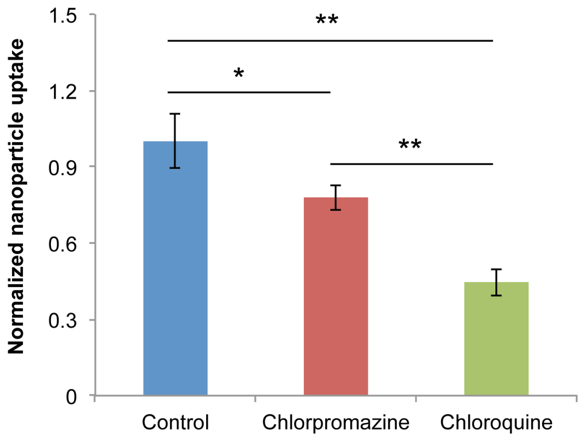

Supplementary Figure 8 | Suppression of liposome uptake in Kupffer cells. Cells were treated with chlorpromazine (30 μM) or chloroquine (100 μM). Chloroquine data from Fig. 2 is included for comparison purposes. Values are normalized to those of control cells. Data is presented as mean ± s.d. of triplicates. Statistics by Student's *t*-test. *, $P < 0.05$; **, $P < 0.01$.

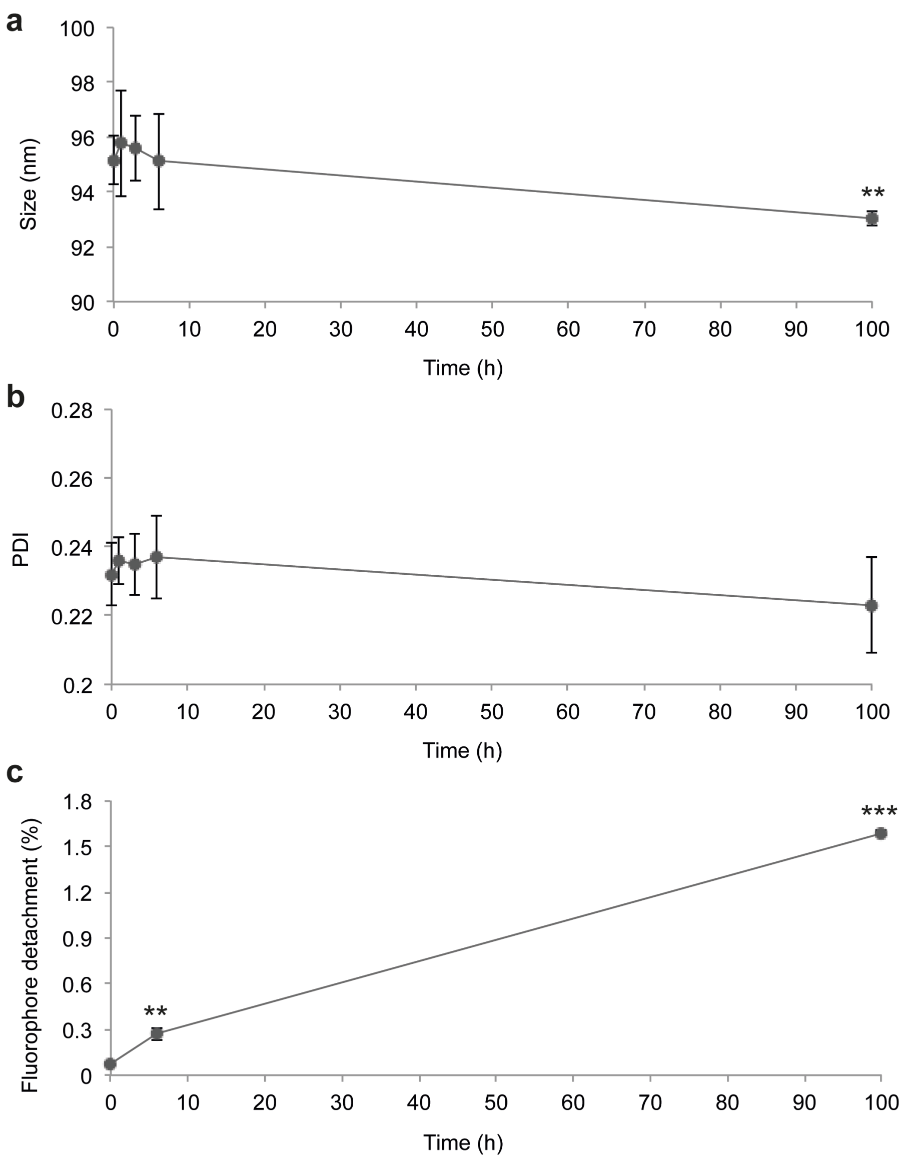

Supplementary Figure 9 | Liposome stability under physiological conditions. Liposomes were mixed with 10% fetal bovine serum (FBS) at 37°C for various time points. The size (**a**) and polydispersity index (**b**) of liposomes were measured by dynamic light scattering. Data is presented as mean ± s.d. of five measurements. (**c**) Fluorophore detachment from liposomes. Statistics by Student's *t*-test. $P < 0.01$; ***, $P < 0.001$, compared to 0 h.

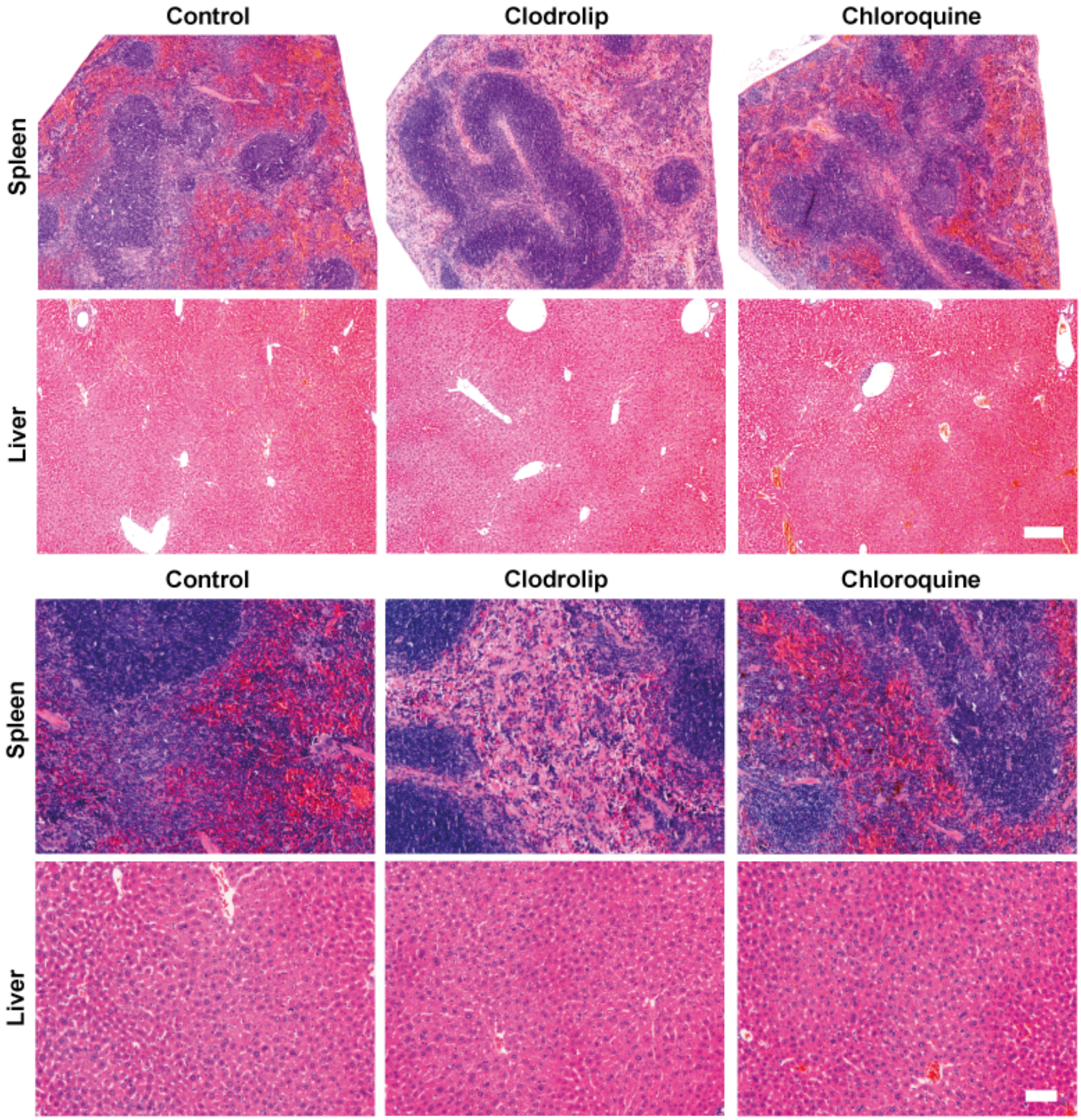

Supplementary Figure 10 | Hematoxylin and eosin (H&E) staining of spleen and liver tissues. Athymic nude mice were intravenously injected with phosphate buffered saline (PBS), clodronate liposomes (clodrolip; 50 mg/kg clodronate for 1 day), or chloroquine (60 mg/kg/day for 7 days). Scale bar, 250 µm (upper), 100 µm (lower).

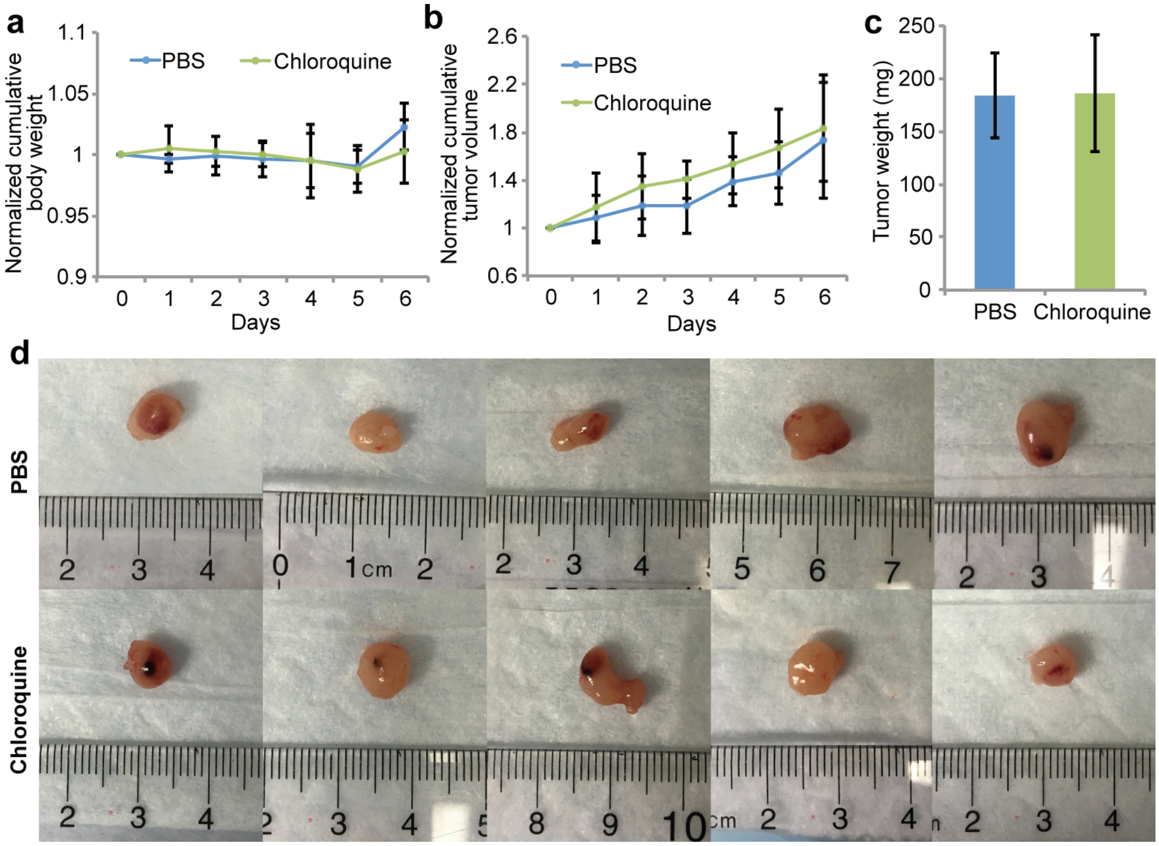

Supplementary Figure 11 | Effect of chloroquine pretreatment on body weight and tumor growth. Athymic nude mice bearing MDA-MB-231 orhtotopic breast tumors were treated with chloroquine for 7 days (60 mg/kg/day). (**a**) Normalized cumulative body weight. (**b**) Normalized cumulative tumor volume. (**c**) Tumor weight at the end of the treatment period. (**d**) Images of tumors at the end of the treatment period. Data is presented as mean ± s.d. (n = 5).

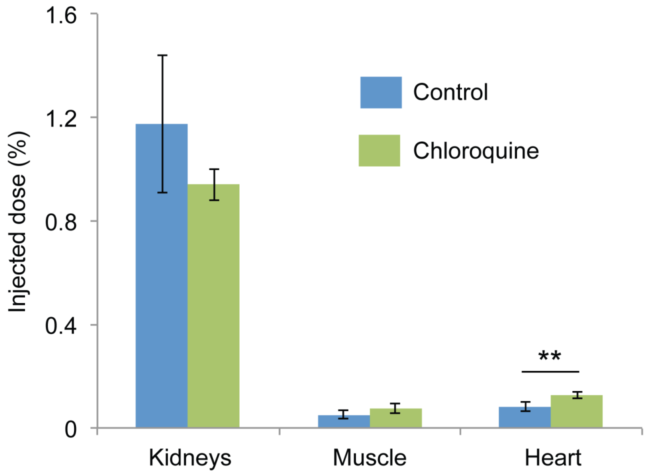

Supplementary Figure 12 | Effect of chloroquine on the accumulation of silicon particles in the kidneys, muscle tissue, and heart. Mice were pretreated with chloroquine (60 mg/kg/day for 7 days). Organs were harvested 15 min after intravenous injection of ^{89}Zr-labeled silicon particles. The dose in 100 µg of muscle tissue is shown. Data is presented as mean ± s.d. of total organ accumulation (*n* = 5). Statistics by Student's t-test. **, *P* < 0.01.

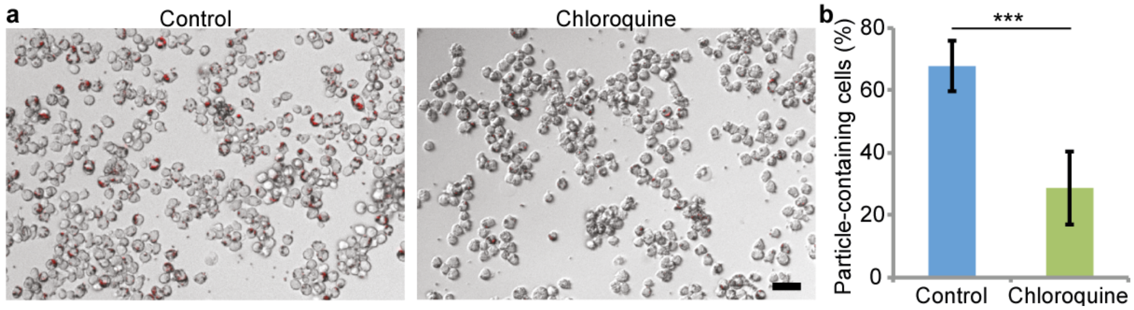

Supplementary Figure 13 | Effect of chloroquine on the uptake of silicon particles in Kupffer cells. Cells were pretreated with chloroquine (100 µM; 30 min) prior to exposure to fluorescently labeled silicon particles (3 h). (**a**) Representative images of cells. Scale bar, 50 µm. (**b**) Quantification of particle uptake. Data is presented as mean ± s.d. of 16 randomly selected regions (20x magnification). Statistics by Student's *t*-test. ***, *P* < 0.001.
